# Medical Items and News of Interest to the Physician

**Published:** 1885-04

**Authors:** 


### Medical Items and News,

### For the Use of Physicians.

CULLED FROM THE STANDARD MEDICAL JOURNALS OF THE WORLD.

NOTE.—These formulæ are intended only for the regular practitioner of medicine, and should be employed only by him, or under his immediate supervision.

**Hemorrhage from the Nose.**

Hemorrhage from the nose usually proceeds from twigs of the facial artery, which are supplied to the structures lining the interior nares, and may almost always be arrested by compressing the alæ firmly between the thumb and finger.

**Irritable Bladder.**

℞

Ammonii chloridi, ʒ ij.

Ammonii bromidi, ʒ iv.

Infus. gentian comp., ℥ vj. M.

Sig.—A tablespoonful three times a day.—*Goodell.*

**Menorrhagia.**

℞
Fl. ext. cannabis indicael, ʒ ij.
Fl. ext. hamamelis virg., ℥ ij.
Syr. aurant. cort., q. s. ad., ℥ iij. M.

Sig.—A teaspoonful four times a day.—*Blackwood.*

**Alcoholism.**

℞

Tr. capsici ʒ iij.

Spts. ammon. aromat. ʒ iij.

Tr. calumbæ, ℥ j.

Tr. card. co., ʒ vj.

Aquam ad., ℥ viij.

M. Fiat Mixtura.

Sig.—A tablespoonful with the same quantity of water every two hours, or when craving for drink comes on.—*Med. Bulletin.*

**Mouth Wash.**

℞

Salicylic acid,

Chlo. potash,

Bi-sulphate soda, aa ʒ j.

Glycerine,

Water, aa ℥ viij.

Ft. sol. M.

Sig.—Teaspoonful to rinse mouth every one to three hours—the mouth being just previously rinsed with plain water.—*Ibid.*

**The Nerve in a Tooth Cavity to destroy.**

After the cavity has been cleaned out, and dried with absorbent cotton, a minute quantity of the following paste is introduced:

℞
Arsenious acid, parts iij.
Morphia sulphate, parts ij.
Creosote, q. s.,
To make a paste.

A small plug of cotton moistened with collodion is then introduced so as to seal up the whole.

**Diabetes.**

Dr. Austin Flint, Jr., adds four more cases of diabetes to the fifty-two reported to the American Medical Association. The patients were placed on strict anti-diabetic diet and Clemen's solution of arsenite of bromide, beginning with three drops, increased to five, was also given. Of these four cases three were permanently relieved. In conclusion he adds: "Diabetes has become to-day a disease easily and certainly curable, provided that the treatment be not begun too late."

**Chronic Bronchitis and Asthma.**

The following is claimed to be a valuable formula:

℞

Potassi iodidi,

Tr. lobelia,

Tr. polygalæ, aa ʒ ij.

Ext. opii, gr. ss.

Aquam distillatum ad., ℥ viij.

M.

Dose—A tablespoonful morning and evening in chronic bronchitis and asthma.

**Leucorrhœa.**

Dr. Fruhauf uses the following method in which the ordinary treatment fails: He commences by disinfecting the vagina by injecting into it a solution containing one to one hundred of carbolic acid and four to one hundred of boracic acid. Then with a speculum, he introduces three or four oakum tampons, each as large as a walnut and containing grs. iss to iij of iodoform, and each having a string attached for ready removal. These are left *in situ* for two or three days. If there is an ulcer of the cervix, he cauterizes it with a solution containing equal parts of acetic acid and distilled water. Of course, in some cases general treatment must be resorted to. For this Fra hauf prefers the phosphate and lactate of iron.—*Jour. de Med. de Paris.*

**Constipation.**

Granzis (*Allg. med. Ctrl. Ztg.*) recommends ergot, in doses of ten grains every two hours, until three doses have been given. This treatment is particularly applicable to cases of constipation depending upon atony of the bowel.

**Mist. Expectorans.**

R

Tinc. sanguinariæ, ℥ iv.

Tinc. opii et camp. Spr. nit. dulc., aa ℥ vj.

Syr. scillæ com. Syr. ipecac. aa ℥ viij.

M. et fit mist.—*Medical World.*

**Nervous Prostration.**

R
Phosphoric acid dil., oz. j.
Elix. calisaya, oz. iv.
Elix. vol. ammon, oz. ij.
Glycerine, oz. iij.
Sherry wine, ad., O j.
Sig.—One to two tablespoonfuls three or four times a day.—*Ibid.*

**Carminative.**

A carminative in infantile colic:

R

Tinct. Assafœtida, 15 drops.

Tinct. Cinnamomi, ½ ounce.

Sodæ Carb, 1 drachm.

Syr. Rhei Aromat, 3 drachms.

Aquæ, 1½ ounces.

M. Sig.: Half teaspoonful every three hours. —*Medical Brief.*

**Hypodermic Use of Caffeine.**

Huchard (*Jour. de med. de Paris*) suggests this combination for hypodermic injection in cases of collapse:
R
Salicylate of Sodium, 45 grains
Caffeine, 1 drachm.
Distilled water, 1½ drachms.
Fifteen minims contain six grains and a half of caffeine.

**Intermittent Fever.**

R

Quiniæ dextro, ʒ ss.

Acid. mur. dil., q. s.

Tr. nucis. vom., ʒ ss.

Ext. tarax. fl.,

Glycerine, aa ℥ ss.

Aq. ad., ℥ ii. M.

Sig.—Teaspoonful four times a day.—*Am. Med. Digest.*

**Night Sweats.**

The *Lancet* gives the following formula for night sweats of phthisis:
R
Sulphate of zinc, gr. iv.
Tinc. of belladonna, ʒ i.
Water, ℥ i.
M. The body to be sponged with the lotion at bed-time.

**Diarrhœa.**

Resorcin, dissolved in castor oil, is a remedy recommended by Dr. Bogresh in the treatment of diarrhœa attended with foul-smelling discharges which contain micro-organisms, and fever with occasional delirium. For adults he gives ℈ i of resorcin dissolved in ℥ v of the oil (warm), to be taken at once [rather heroic?], and to children, gr. viij, each, of the resorcin and oil.

**Headache.**

Brassicon is a mixture much in use in the province of Kief for headache. It is compounded of—
R
Oil of peppermint, grs. xxx.
Oil mustard volatile, gutt. vj.
Camphor, grs. x.
Ether, ʒ j.
Alcohol, 90 per cent., ʒ iij.
Tincture of balm, q. s. to color.
—*Ph. Z. f. Russ.*

**Pilocarpine.**

Regarding the beneficial results attending the local sweating with pilocarpine in local troubles, Dr. Baux places this method of medication at the head of all other known remedial measures. In thirty-one cases of fractures, falls, torticollis, luxation, arthritis, articular rheumatism, and other affections, especially of bones and joints, he has obtained a degree of success respecting rapidity and completeness of cure unattainable in his opinion by other forms of treatment. In hydrarthrosis, pilocarpine has no curative effects; it proved useful, however, in two cases of white swelling, in cases of orchitis and of inflammatory swelling after injection of tincture of iodine into the tunica vaginalis.

Regarding the mode of its application, Beaux considers its endermic use, *i. e.*, after vesication of the cutis, as the most effective method of producing absorption of the drug.—*These inaug.*, Lyon.

**Iodoform Eruption.**

Neisser reports six cases of eruption, of impetiginous character, following the external use of iodoform. Relief followed the stopping of the iodoform and the application of a 5 per cent. solution of acetate of aluminum.

**Communication of Disease by Insects.**

In a communication in the *Cronica Med.-Quirurgica de la Habana*, Dr. Finley states that he found that mosquitos which had bitten a yellow-fever patient carried away with them the filaments and spores of a peculiar nature, which were especially found upon the proboscis. He thinks it fair to conclude that disease may be carried in this way, although he does not look upon it as the only method of communicating yellow-fever.—*Medical Times.*

**Calendulated Boracic Acid.**

For chronic suppuration of the middle ear, Dr. Prout, of Brooklyn, N. Y., recommends the following:

℞

Tincture of calendula, f ℥ j.

Boracic acid in finest powder, ℥ j.

Mix and expose for evaporation. When fully dry, add to the above:

Boracic acid ℥ ij, and rub into a very fine powder.

To be blown into the ear after dry cleaning. —*Proc. Med. Soc., Kings Co.*

**Rheumatism and Diet.**

Rheumatism is, as often as not, caused by over-eating, and especially over-indulgence in meat, which is certain to cause an excess of uric acid, and render the body liable, on exposure to wet or cold, to an attack. We know that old people are proverbially liable to rheumatism. The reasons for this are not far to seek. One is that joints and ligaments are harder and stiffer, and very often contain a deposit—urate of soda. Another is that, as a rule, people up in years eat more than is necessary to suppórt life, under the mistaken notion that they want a deal of nourishment to keep them up. I say that, on the contrary, the wear and tear of tissue is trifling compared to what it is in earlier manhood, and that far less food is required. Therefore, if an elderly person would live long, and be free of aches and pains, and be calm in mind—for that is a great desideratum—he or she must live abstemiously, more or less.—*Cassell's Magazine.*

**Chronic Asthma.**

Apomorphine—Dr. Urber, of Darmstadt, relates a case of chronic asthma (thirty years' standing) which was permanently cured in three weeks by the use of apomorphine in doses of ½ grain increased to ⅕ grain, thrice daily.

**Phthisis.**

According to the Lancet, Dr. Pick affims that aluminium and its compounds constitute a most effective remedy against pulmonary tuberculosis. His statement is based upon results noted in experiments upon rabbits, as well as on clinical observations. In one case where infiltration of the apices of the lungs had occurred, the morbid symptoms are said to have disappeared on the use of the following compound: Metallic aluminium, grammes (℥ ij); aluminium hydrate, 5 grammes (℥ i½); calcium carbonate, 5 grammes (℥ j½); gum tragacanth q. s. This was divided into 60 pills, one of which was administered three times a day.

**Cholera Studies.**

The newspaper report that Dr. Koch had finally succeeded in inoculating rabbits with cholera by his comma bacilli is not true. Dr. Koch has not yet succeeded in producing cholera in the lower animals by means of his cholera bacilli, or if he has, the report has not been made public.

On the contrary, the investigations of the British cholera commissioners, Drs. Klein and Gibbs, have so far, according to the *British Medical Journal*, tended to confirm the view that comma bacilli are to be found in other diseases than Asiatic cholera. Futhermore, the gentlemen referred to have so far found that the bacilli are not present in great numbers in the intestines of those who die suddenly of cholera. Still more important is the fact reported that the comma bacilli of cholera and of other diseases develop in much the same way, and that there is nothing specific in the mode of growth of Koch's bacillus.

Drs. Finkler and Prior, who discovered a comma bacillus in cases of cholera nostras, are continuing their researches in the laboratory of Professor Ceci at Genoa. It is quite well determined now that their comma bacillus does not develop in the same way as Koch's, and that it is of a slightly different form.—*Med. Record.*

**Hoarseness in Speakers and Singers.**

M. Corson advises the placing in the mouth of a piece of borax, about two or three grains; it produces an abundant salivation, and the voice becomes clear. He also recommends the use of a couple of grains of potasium nitrate in a glass of sugar and water, or an infusion of forty-six grains of jaborandi, and—shortly before using the voice—of a gargle with six or seven ounces of a decoction of barley, one to two drachms of alum, and two drachms of honey of roses.

**Sciatica.**

To afford immediate relief to the patient I charge a syringe with eight drops of chloroform and introduce the needle its full length into the muscles of the ilio-femoral crease. In two days this application will be repeated, while the man will at once be put on the use of strong lemonade.

Here a very practical experience is to be kept in memory: Hypodermic injections of chloroform, while affording relief in sciatica, acts exactly conversely in facial neuralgia.—*Garretson.*

**Piles.**

The following ointment will cure most cases of piles, even those that would seem to require an operation. A number of cases of chronic prolapsus ani have yielded to it:

℞

Monselle's Salt, ½ ounce.
Morph. Sulph., 6 grains.
Petroleum Mass., 1 ounce.

Be sure to use petroleum mass; lard, vaseline, etc., should not be substituted, as the crude petroleum has a specific effect on the parts diseased.—*Medical Brief.*

**Acute Alcoholism.**

The following mixture is in use in the Albany Hospital for the treatment of the effects of acute alcoholism, to relieve nervous excitement and insomnia:

℞

Tr. opii. deod,

Ext. hyoscyam fld, aa ʒ j.

Chloral hydrat,

Pot. bromidi, aa ʒ j.

Tr. capsici, ʒ ss.

Tr. aconiti rad, ♏ v.

Aqua menthæ pip,, ad., ℥ iv.

M. Sig.: Two tablespoonfuls and repeat in four hours if sleep is not produced.—*Medical Summary.*

**Snake Bite.**

You will find the seeds of the blessed thistle a prompt and unerring antidote in nutralizing the infection of all poisonous reptiles, and especially that of the rattle-snake. Give three seeds (bruised) every ten minutes, in sweet milk, until relief is obtained. In most cases nine seeds will relieve all apprehension for the patient's safety. Cockleburrs are used as a domestic remedy by the use of strong decoctions.—*A. J. Shæffer, M. D., in Med. Brief.*

**Locomotor Ataxia.**

Eulenberg ("Lyon med."; "Glasgow Med. Jour.") has treated three hundred cases with hypodermic injections of silver, using the following formula:

Chloride of silver, 2 grains;
Hyposulphite of sodium, 10 grains;
Distilled water, 5 drachms.

The injections are made in the back. The patient is said to obtain a rapid relief from pain, which, however, is only temporary. Besides silver, the writer uses local applications of cold, the constant current, and hypodermic injections of strychnine.

**Nasal Polypi.**

Dr. William Ralph Bell says: "I take the liberty of bringing the mode of treatment before the notice of your readers which I have practiced with the very best results in several cases. It obviates any trouble from hemorrhage, which is frequently the case when the forceps or hooks is used; it is painless and very simple. I get my patient to blow strongly through the affected nostril, closing the other with his finger. The polypus will be brought down so that it can be easily seen through the external nares; then, with my hypodermic syringe charged with a solution of tannic acid in water (of the strength of twenty grains to the fluid drachm) I pierce the polypus with the needle, and inject ten, fifteen or twenty minims of solution, according to the size of tumor. In a few days the polypus shrivels and drys up (tanned); it comes away without any trouble or pain, aud looks like a clot of dry blood, my patients usually removing it by blowing the nose or by their fingers. In only one case, that of an old lady, had I occasion to remove it myself, and in her case I think she was afraid to do so, for when I seized it with dressing forceps, I required to make no traction to bring it away."—*Canada Med. Record.*

**Tape Worm.**

Professor Palmer, of Michigan University, prescribes kousso, which is a most efficient tæniafuge, in the following form:

℞

Eth. ext. male fern ℥ ss.

Pulv. kousso ℥ ij.

Confec. senna, q. s. to make pills iij.

He says that the patient should abstain from food for twelve hours, then take one of these pills every hour, followed by a dose of castor oil.—*Druggists Circular*.

**Diarrhœa.**

Despres (*Ibid.*) is credited with the following formula:

℞

Chloroform, 15 grains.

Alcohol, 2 drachms.

Solution of acetate of ammonium, 2½ drachms.

Syrup of hydrochlorate of morphine, 10 drachms.

Water, 3½ ounces.

Dose, a tablespoonful every half-hour.

**Colic of Infants.**

In flatulent colic of infants, I have found the following formula almost a specific: Take

Bromide of potassium, 1½ drachms.

Glycerine, 4 drachms.

Water, 1½ drachms.

Misce. Sig.: A teaspoonful every half-hour until relieved.

The dose of bromide of potassium for a child one year old is from three to ten grains, repeated every two or three hours, in solution. In spasmodic conditions, it should always be given in sufficient doses to produce its full physiological action.

**Liniment for Man or Beast.**

℞

Ol. cloves, f ʒ i.

Ol. spruce, f ʒ i.

Ol. cinnamon, f ʒ ½.

Spt. ammonia, f ʒ i.

Spt. turpentine, f ʒ v.

Gum camphor, ℥ ii.

Alcohol, Oj.

Vinegar, Oj.

Chamber-lye, Oj.

Boil down the vinegar and chamber-lye to one pint, and then add the other ingredients. —*A. C. Russell, M. D., West Mills Grove, N. Y.*

[For a cheaper "liniment" and one just as good, go to the nearest cess-pool.—ED.]

**Syhilitic Ulcers.**

Terillon (*Union Med.*) gives the following formula:

℞

Pyrogallic acid, 5 drachms.

Starch, 5 drachms.

Vaseline, 2 ounces.

This ointment is to be spread upon charpie, and applied to the ulcers once a day. A second application is not generally necessary, unless the leisons are very extensive. For this preparation a powder composed of equal parts of pyrogallic acid and starch may be substituted.

**Coughs.**

Coughs differ so much from each other in causation that it is very difficult to give any home prescription. In an adult, the feet may be soaked in hot mustard water, a mustard plaster applied to the chest, or the chest rubbed with a mixture of sweet oil to which a little croton oil has been added. Or:

℞

Paregoric, gutts. xv.

Aqua, a wineglassful.

Mix. Take about every three hours. Or:

℞

Ammon carb., grs. xv.

Syr. seneg., ʒ jss.

Syr. prun. virgin, ℥ jss.

Syr. tolu, ʒ ij.

Aqua, f ℥ jss.

Mix. Dose, a tablespoonful every two or three hours.

An excellent domestic remedy for a cough is:

℞

Hoarhound leaves, ℥ j.

Water, 1 pint.

Boil for fifteen minutes, strain out the leaves, add enough sugar to make nice syrup, boil for fifteen minutes. The dose is a tablespoonful every hour.

It is to be constantly borne in mind that the early stages of many severe diseases, as pneumonia, typhoid fever, small pox, etc., are ushered in by symptoms which are not readily distinguished from those of an ordinary cold. It is a good rule in all cases to take the best possible care of the body in health, and then, whenever it is deranged, to call in the family physician at once, and implicity follow his advice. A cold may be a little thing, but it often leads to serious disorders.

**Laryngitis.**

Resorcin is recommended by Andeer as a local application, on account of its action as a disinfectant and local anæsthetic. Strong solutions are caustic, while dilute ones are merely astringent.

**Laxative.**

R

Ext. nux vom, gr. ¼

Ex. belladon, gr. ⅙

Comp. ex. colocynth, gr. ij

Powd. Socot aloes, gr. j.

M.—Make 1 pill.

**Sleeplessness.**

Of the new hypnotic the *Medical Press* says: Paraldehyde is claiming the attention of the profession, as in some respects it is superior to chloral, and is very safe to give in simple sleeplessness, and leaves no unpleasant after effects. However, it is of no use when insomnolency is the result of pain. It is given in the following formnla: Take

Paraldehyde, 1 drachm.

Syrup of orange, 1 ounce.

Water, 1 ounce.

To be taken at bedtime—*Medical News.*

**Gout.**

In all forms of gout Dr. Granville has given the following formula with such success that he recommends its trial by others. He never gives colchicum.

R.

Ammonii chloridi, ʒ iv.

Potassii chloratis, ʒ ii.

Glycerin, f ʒ xii.

Tincturæ iodini, f ʒ ii.

Aquam ad, f ℥ xii.—M.

Dose: Two tablespoonfuls four to eight times daily.—*Lancet.*

**Phthisis and Asthma.**

According to Dr. George F. Hawley (*Western Medical Reporter*,) mullein has been used quite extensively in the St. Vincent Hospital, Dublin, in the treatment of phthisis and certain forms of asthma. It is given in the following manner: one ounce of the dried leaves is added to a pint of milk which is placed over the fire till it reaches the boiling point, when it is strained through a coarse cloth. This is to be taken during the day, one-half in the morning and the rest at night. In cases of diarrhœa it seems to check the discharge. The leaf may also be smoked and the smoke inhaled in cases of asthma in the form of a cigarette, a little tobacco being added if unpleasant to the taste. Its use is said to be followed by disappearance of dyspnœa.

**Tinea Capitis.**

R

Acid pyrolig., 1000 gr.

Hydrarg. oxid. rub,, 2 gr.

Acid salicylic, 1 gr.

M. To be carefully applied. It may be necessary to apply it at intervals of some days for five or six times.—*Journal de Med.*

**Lupus Vulgaris.**

I have for some time employed salicylic acid, in the form of ointment, as a remedy for eczema of the scalp, and impetigo contagiosa occurring in children, with the most satisfactory results, cases that have defied other treatment yielding rapidly to its agency, and I have been induced to make a further trial of it in other skin affections.

By the kindness of Mr. Rigby, surgeon to the Doncaster Infirmary, I was permitted to employ it in a very bad case of lupus exedens, which had been for some time under his care.

The patient, a woman about 25 years of age, had her face terribly disfigured, the ulceration having destroyed one *ala nasi*, the whole of the cheek and eyebrow being also involved. She had been in the hospital before, and had improved under treatment with Donovan's solution and a visit to Harrogate. But on her return, although she was kept under observation and treatment, fresh tubercles developed, and the parts that had been cicatrised soon became again involved, and she was re-admitted to the institution. I first tried the ointment of 15 grains to the ounce of vaseline, which was of no use; I then increased the strength to a drachm, and then to a drachm and a half to the ounce.

The ulcers soon began to heal, no fresh tubercles appeared; the cicatrices became soft and lost their shiny, unhealthy appearance, and the skin of the face is now almost sound. She was taking a mixture containing Donovan's solution and the liquor ferri dialysti. But as this had been administered without apparent benefit before admission, I think it is fair to give the credit to the internal remedy. I have not heard of salicylic acid being employed before in the treatment of this particular disorder, and its action seems very satisfactory, especially as it does not appear to cause irritation.—*J. G. Marshall, M. B. Cantab., M. R. C. S., in the Brit. Med. Journal.*

